# Sustainable Solid-State Sodium-Ion Batteries Featuring Ferroelectric Electrolytes

**DOI:** 10.3390/ijms252312694

**Published:** 2024-11-26

**Authors:** Ângela Freitas, Manuela C. Baptista, Maria Helena Braga

**Affiliations:** 1 Department of Engineering Physics, Faculty of Engineering, University of Porto, Rua Dr. Roberto Frias, 4200-465 Porto, Portugal; up201906619@edu.fc.up.pt (Â.F.); mbaptista@fe.up.pt (M.C.B.); 2 MatER, Faculty of Engineering, University of Porto, Rua Dr. Roberto Frias, 4200-465 Porto, Portugal; 3 LAETA, Faculty of Engineering, University of Porto, Rua Dr. Roberto Frias, 4200-465 Porto, Portugal

**Keywords:** sustainable battery, solid-state, sodium ion, glassy ferroelectric electrolyte, electrodeless, self-charge, DFT simulation

## Abstract

Solid-state batteries offer significant advantages but present several challenges. Given the complexity of these systems, it is good practice to begin the study with simpler models and progressively advance to more complex configurations, all while maintaining an understanding of the physical principles governing solid-state battery operation. The results presented in this work pertain to cells without traditional electrodes, thus providing a foundation for guiding the development of fully functional solid-state cells. The open circuit voltage (OCV) of the Cu/Na_2.99_Ba_0.005_ClO composite in a cellulose/Zn pouch cell achieves 1.10 V, reflecting the difference in the chemical potentials of the current collectors (CCs), Zn and Cu, serving as electrodes. After 120 days, while set to discharge, conversely to what was expected, a higher potential difference of 1.13 V was attained (capacity of 5.9 mAh·g^−1^_electrolyte_). By incorporating a layer of carbon felt, the OCV became 0.85 V; however, after 95 days, the potential difference increased to 1.20 V. Ab initio simulations were additionally performed on a Cu/Na_3_ClO/Zn heterojunction showing the formation of dipoles and the Na deposition on Zn which is demonstrated experimentally. The sodium plating on the negative CC (Zn) takes place as the cell is set to discharge at room temperature but is not observed at 40 °C.

## 1. Introduction

Considering the alarming increase in global temperatures, the necessity for innovative and sustainable energy solutions has reached a critical point. Renewable energy sources, which utilize naturally replenishing resources and produce minimal greenhouse gas emissions, represent a promising avenue for addressing this challenge [1,2,3]. However, these energy sources are contingent upon specific conditions to generate electricity and thus are not consistently available. Consequently, there is an increasing need for renewable energy storage devices that can store energy to utilize during periods of limited production [4,5,6].

The field of solid-state batteries is advancing rapidly, achieving new maturity levels [7,8,9]. This progress is primarily driven by the efforts of major companies, specializing in this technology, to expedite the launch of their first commercial solutions [10]. As a result, vehicle manufacturers are accelerating their plans to extensively incorporate this technology [11,12]. Solid-state batteries address several limitations of lithium-ion batteries (LIB), such as slow charging rates, safety problems due to flammable liquid electrolytes, dendrite formation, thermal runaway and oxygen release and restricted operation at high temperatures (above 40 °C), among others [13,14,15].

Sustainability strategies have become a focal point in the battery industry, compelling manufacturers to adopt more responsible practices [16,17]. This change highlights the industry’s reliance on cobalt and the challenges associated with its high cost and limited availability [18]. Consequently, it is vital to examine the value chain of critical materials and develop strategies that effectively address these challenges [19,20,21]. Sodium has emerged as a leading candidate for replacing lithium, as both metals are alkaline with similar properties, with sodium being a thousand times more available than lithium [22,23,24,25,26]. This similarity enables easier optimization of sodium-ion batteries (SIBs), leveraging the extensive research already conducted on lithium. Moreover, sodium’s abundance significantly reduces its cost [27]. Additionally, SIBs can be more cost-effective due to sodium’s compatibility with aluminum CCs, which are inexpensive and lightweight [28,29]. However, it is essential to evaluate selected materials to maximize the potential benefits of SIBs, as LIBs show higher energy density [30]. Typically, the SIB shows a gravimetric energy density of 100–160 Wh·kg^−1^ and an energy density of 200–400 Wh·L^−1^ and the LIB 150–250 Wh·kg^−1^ and 400–700 Wh·L^−1^, respectively. A comprehensive approach to technological exploitation, covering everything from material selection and synthesis to processing and end-of-life reclamation is necessary prior to changing the currently adopted technology [31,32,33].

Research on solid-state batteries has been ongoing for over a decade, with a consistent focus on designing high-performance, safe, and economical electrolytes that are also compatible with optimized electrode materials [34,35]. A study from 2014 introduced an innovative material, a new amorphous electrolyte evolving from an antiperovskite structure demonstrating the highest ionic conductivity ever reported for lithium batteries, 25 mS·cm^−1^ at 25 °C [36]. In summary, crystalline antiperovskite precursors A_3−x_H_x_ClO (where A = Li or Na and 0 < x < 1) can be converted into Li^+^ or Na^+^ glassy electrolytes by adding water up to their solvation limit, to then extracting it at 230–250 °C, while obtaining a dry solid electrolyte. Given the advancements in battery technology in recent years, these studies were particularly visionary in exploring sodium glassy electrolytes [37,38].

Considering that the electrolyte is one of the fundamental components of a cell, an immediate question emerges: *What properties distinguish these glassy electrolytes from other solid-state electrolytes?* Ferroelectric solid electrolytes, which may accommodate multiple dipoles cooperatively ordered below a certain transition temperature, exhibit pyroelectric properties. All pyroelectrics are inherently piezoelectric. This relationship seems to extend to amorphous solids containing ferroelectric molecules [39].

**Figure 1:**
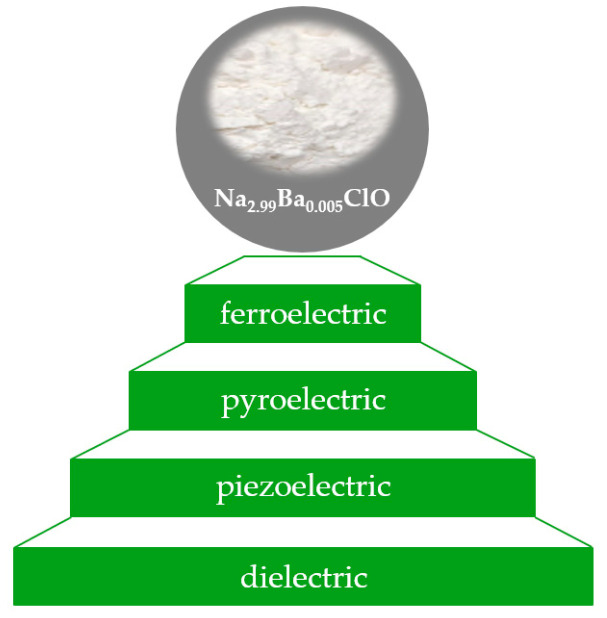
Relationship between dielectric, piezoelectric, pyroelectric and ferroelectric materials. The morphology of Na_2.99_Ba_0.005_ClO the ferroelectric electrolyte, as synthesized.

In the dielectric materials world of electronic insulators, a smaller set of materials can be polarized when subjected to applied stress (piezoelectric) and an even smaller set when subjected to a temperature rate (pyroelectric) [39,40] (Figure 1).

To better understand the behavior of this material, it is necessary to study the polarization of the glassy material. The polarization can occur due to (i) a shift of the atomic electronic charge relative to the positive nuclear charge, creating a dipolar axis parallel to the applied electric field (***E***_a_); (ii) a shift of cations along the crystal axis from the center of symmetry of their site in an ionically bonded crystal, resulting in a polarization component parallel to ***E***_a_; or (iii) an orientation of intrinsically dipolar molecules (e.g., A_2_O or OA^−^, A = H, Li, and Na) to increase the component of molecular dipoles parallel to ***E***_a_. Therefore, the spontaneous polarization of a ferroelectric electrolyte (polarization at zero potential) increases the cell’s capacity [41].

Ferroelectric energy storage cells with glassy electrolytes demonstrate self-charging and self-cycling behaviors [13]. Knowledge of surface transport is essential from a fundamental point of view. In this sense, in 2021, the tendency of Li_3_ClO, Li_2.92_Ba_0.04_ClO, Na_3_ClO, and Na_2.92_Ba_0.04_ClO ferroelectric-electrolytes to maintain phonon oscillation coherence during a short time-lapse in (ps) was shown, contributing to the polarization of the ferroelectric [42].

In the recent pursuit of innovative energy solutions, ferroelectric topological insulators (FETIs) have emerged as promising candidates [43,44,45,46,47]. The synchronization of surface potential (voltage) oscillations, temperature, and mass with volume oscillations enables the harnessing and storage of electrical energy within a single FETI or as a part of a battery [48]. This capability offers a wide range of potential applications, including wireless batteries, transistors, memories, sensors, and selective catalysts [48].

This family of electrolytes enables the exploration of batteries with various configurations from full to anode- or electrode-less cells fabricated as coin, pouch, and structural cells. Coin cells are inherently the testing cells because of their compact size [49]. The pouch-type cells are particularly investigated for their design flexibility, reduced weight and volume, improved heat dissipation, and closer proximity to application formats [50,51,52]. However, there is also growing research in structural batteries due to their potential for integration into various structures where spatial efficiency is not crucial, while multifunctional capabilities are necessary [14,53] such as mechanical reinforcement beyond energy storage.

In the context of sustainability, it is essential to highlight the concept of a circular economy [54,55]. The recycling of materials with lower economic value but greater environmental sustainability, such as LiFePO_4_, presents an economic challenge [56]. To address this challenge, studies are investigating the recovery of FePO_4_ electrodes from aged Li-ion for reuse in Na-ion battery cells [57]. This represents a significant step towards enhancing sustainability [58,59].

This work demonstrates the possibility to use pouch cells (PC) containing a Na_2.99_Ba_0.005_ClO electrolyte. The ferroelectric sodium-ion electrolyte was fabricated as a separator composite and utilized between zinc and copper foils serving as the negative and positive CCs, respectively. These cells were enhanced by incorporating a sheet of carbon felt adjacent to the positive CC, yielding superior performance. The interfaces and crystal structure as well as the electron localization functions (ELF) were simulated to identify dipoles and Na-plating features and compare with experimental analyses.

## 2. Results and Discussion

This section presents electrochemical and morphological data for the cells in Table 1; additional details are given in Section 3.

### 2.1. Performance of Cu/Na_2.99_Ba_0.005_ClO Composite in Cellulose/Zn Pouch Cells

The pouch cells consisted of two CCs: (1) a copper foil serving as the positive electrode, and (2) a zinc foil as the negative electrode separated by (3) a solid ferroelectric electrolyte of Na_2.99_Ba_0.005_ClO mixed with PVA_C_ impregnated in cellulose.

#### 2.1.1. Heterojunction: Ab Initio Simulated Interfaces

To simulate the cell, a couple of simplifications were established: (1) a limited number of atoms: 87 atoms were used including (Cu)_52_, (Zn)_20_, and (Na_3_ClO)_3_; (2) no inclusion of Ba, as it would imply using many more atoms to achieve the Na_2.99_Ba_0.005_ClO stoichiometry and (3) no inclusion of PVAc and cellulose. These simplifications do not invalidate the conclusions as Ba reinforces the ferroelectric behavior perceived in these simulations for Na_3_ClO, in accordance to previous simulations [42]. The PVAc and cellulose role is not related to the ferroelectricity of Na_3_ClO or to sodium plating, therefore conferring a broader significance to these simulations.

**Figure 2:**
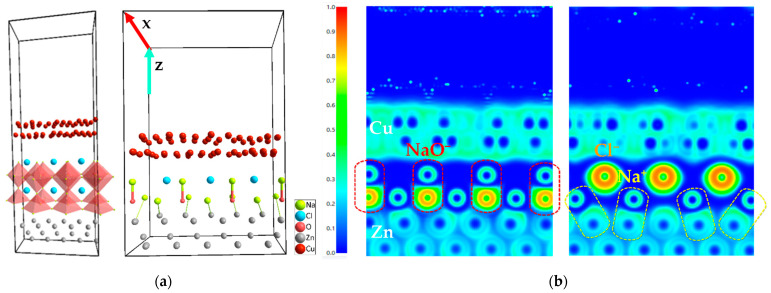
Heterojunction Cu/Na_3_ClO/Zn; simulated structures showing (**a**) the **NaO^−^** dipoles and **Na^+^ deposition** on Zn optimized after simulating the Cu/Na_3_ClO and Na_3_ClO/Zn interfaces; (**b**) ELF showing NaO^−^ dipoles (**left**); “free Cl^−^” and Na^+^ deposited on Zn parallel to the Zn-surface direction (**right**) which may be indicative of the Zn quasi-parallel Na-plating direction observed in SEM images. **Note:** ELF = 1 and ELF = ½ correspond to localized electrons and electron gas, respectively.

The interfaces Cu/Na_3_ClO and Na_3_ClO/Zn were simulated and then integrated to form a Cu/Na_3_ClO/Zn cell, which was subsequently optimized using DFT allowing the atom positions and shape to relax. The simulated heterojunction (Figure 2a) does not actually represent a cell where the parts are individually synthesized and then assembled as in traditional pouch-cell fabrication, but a sputtered cell formed by atomic deposition, where each atomic layer is deposited on the previous.

Figure 2b shows NaO^−^ dipoles (left) aligning with the oxygen towards the Zn surface between two Na-ions that will eventually plate as Na metal (right). The two simulated electron localization function (ELF) images correspond to the [100] Miller indices at x = 3.85227 (left) and x = 1.25940 (right), respectively.

Although the copper seems to be positioned very far from the Na_3_ClO, the ELF shows that the electronic cloud of the Cl^−^ repeals the Cu^2+^ sea of free electrons. In the zinc, most electrons are localized. Highlighted in Figure 2b (right), the natural alignment of the bond between the sodium and zinc is shown, which may explain the SEM/EDX images of the plated sodium shown hereafter where the Na grows quasi-parallel to the zinc surface eventually covering it.

The formation of dipoles is essential to the performance of the cell. Dipoles align their negative charge to the copper side, at the lower absolute chemical potential, and the positive to the zinc, at the higher absolute chemical potential. This alignment has the following advantages for the working cell: (1) equilibrating the electrochemical potential of Na_3_ClO at both interfaces achieved through the dipole ends at a lower energetic cost; (2) the charge transfer is made through the dipoles; (3) the ionic displacement to reverse polarization becomes smaller, as it is achieved by losing or adding a Na^+^-ion; (4) more charge may be accumulated at the electrodes as the ferroelectric possesses a higher dielectric constant than the traditional electrolytes; (5) the Na^+^-ions grow in excess on the Zn side, facilitating Na-metal plating while charging the cell.

#### 2.1.2. Electrochemical Cycling

**Figure 3:**
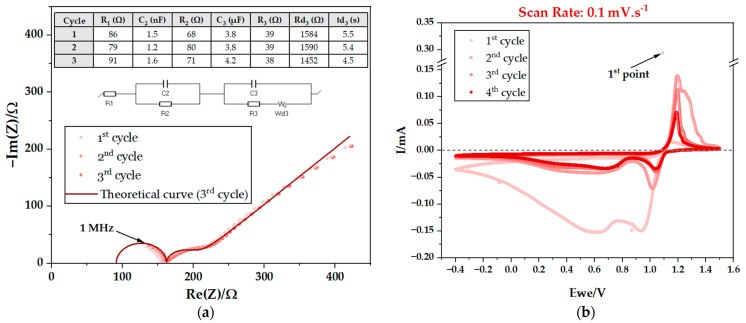
Electrochemical performance of a Cu/Na_2.99_Ba_0.005_ClO composite in cellulose/Zn pouch cell (PC I): (**a**) 1st PEIS before performing CV; (**b**) 1st CV scan rate: 0.1 mV·s^−1^.

**Figure 4:**
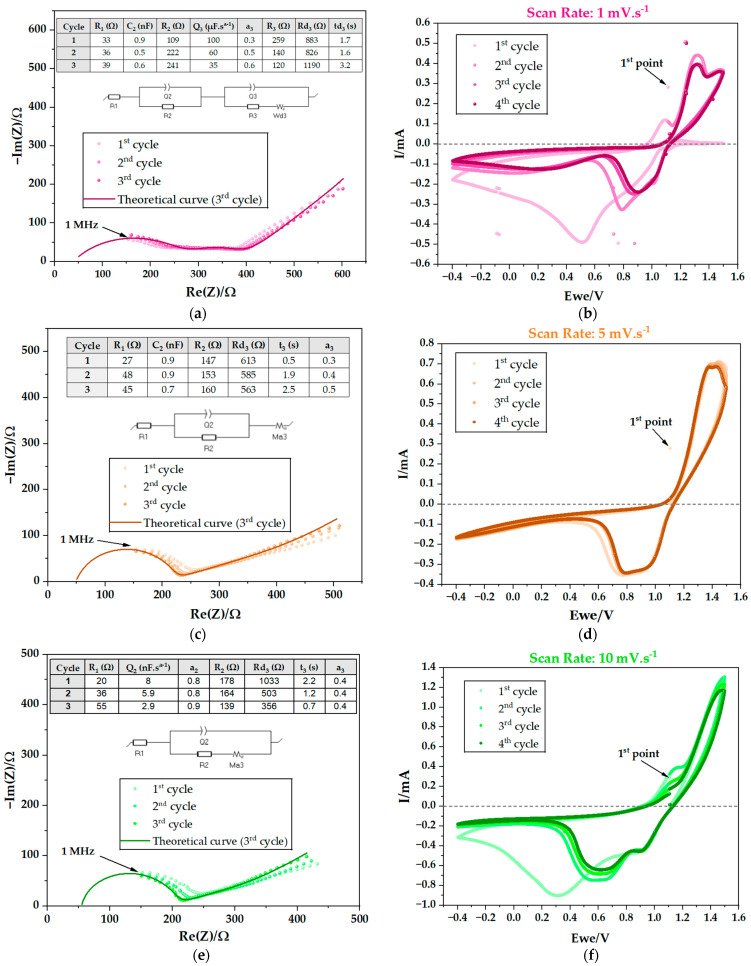
Electrochemical performance of a Cu/Na_2.99_Ba_0.005_ClO composite in cellulose/Zn pouch cell (PC I): (**a**) 2nd PEIS after 1st CV; (**b**) 2nd CV scan rate: 1 mV·s^−1^; (**c**) 3rd PEIS after 2nd CV; (**d**) 3rd CV scan rate: 5 mV·s^−1^; (**e**) 4th PEIS after 3rd CV; (**f**) 4th CV scan rate: 10 mV·s^−1^.

**Figure 5:**
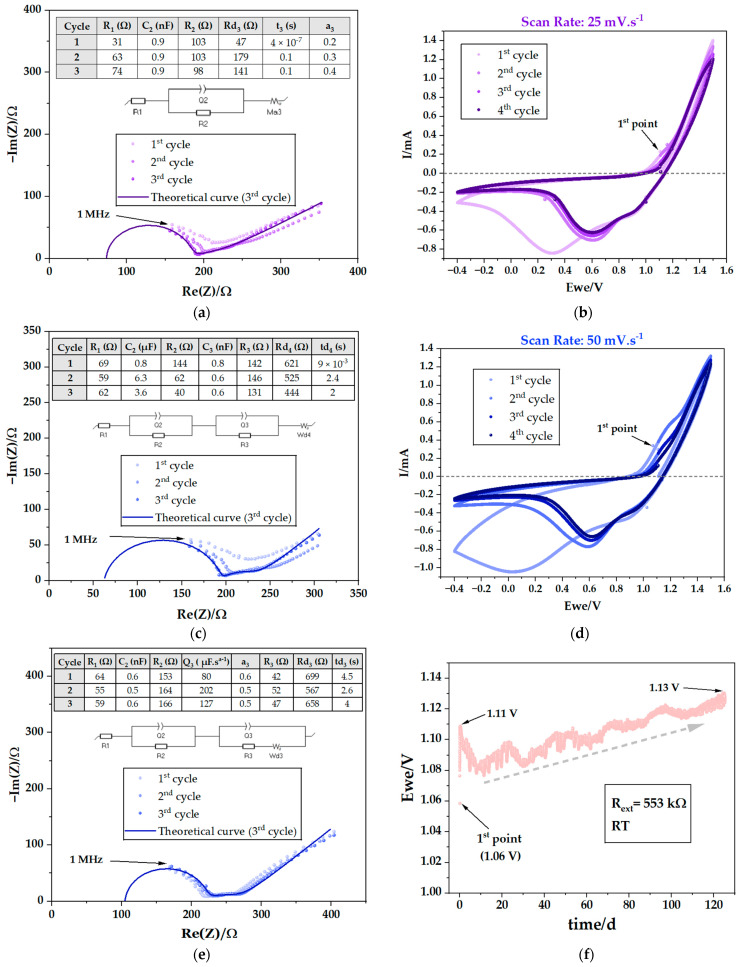
Electrochemical performance of a Cu/Na_2.99_Ba_0.005_ClO composite in cellulose/Zn pouch cell (PC I): (**a**) 5th PEIS after 4th CV; (**b**) 5th CV scan rate: 25 mV·s^−1^; (**c**) 6th PEIS after 5th CV; (**d**) 6th CV scan rate: 50 mV·s^−1^; (**e**) 7th PEIS after 6th CV; (**f**) discharge with an external resistance of 553 kΩ associated with the cell at room temperature.

Immediately after assembly, the pouch cells underwent electrochemical testing. Potentiostatic electrochemical impedance spectroscopy (PEIS) was conducted, followed by cyclic voltammetry (CV) at various scan rates of 0.1, 1, 5, 10, 25, and 50 mV·s^−1^, combined with additional PEIS (Figure 3, Figure 4 and Figure 5). This study was conducted on PC I (Table 1). The results obtained by PEIS were then analyzed using equivalent circuits. By interpreting the Nyquist diagrams in PEIS, it is possible to extract detailed information about the mechanisms and features of the electrochemical systems (Table 2) including the resistance of the bulk electrolyte and, therefore, its ionic conductivity. It is also possible to analyze the charge transfer resistance associated with the electrolyte/CC interface (Zn and C or Cu). Furthermore, the double-layer capacitance, reflecting the charge storage capacity at the interfaces, may also be determined by PEIS.

As highlighted before, it is possible to determine the ionic conductivity (σ) using Nyquist plots; in spectroscopy, each semicircle corresponds to resonance with a different type of movement of a species. The highest frequency corresponds to the highest mobility. In the present case, the highest frequency semicircle corresponds to the resistance to the ionic movement of the bulk ions in the electrolyte represented by the equation $\sigma =\frac{1}{R}\left(\frac{d}{A}\right)$, where R represents the ionic resistance, while d and A represent the thickness and the cross-sectional area, respectively. The Na^+^ ion is the mobile element in the Cu/Na_2.99_Ba_0.005_ClO/Zn capacitor with the highest natural frequency within the working range of [1 MHz–0.1 Hz].

The highest frequency semicircle is represented by an equivalent circuit composed by an association in parallel between a capacitor with capacitance C_1_ and a resistor with resistance R_2_, which is in series with R_1_ (Figure 4c,e and Figure 5a). R_2_ is the resistance of the capacitor thickness d and surface area A, where the bulk ions are freer in the cell, not affected by the coulombic forces due to the accumulation of ions or vacancies at the interface. The R_1_ represents electrical and ionic resistive phenomena, independent for each species and interface of the cell; in other words, phenomena that do not affect both surfaces of the electrolyte and electrodes at the same time.

Additional semicircles may be shown at lower frequencies representing phenomena at the interfaces such as the natural formation of electrical double layer capacitors (EDLC) to compensate for differences in chemical potentials between electrolyte/electrode. A second semicircle corresponds to an association in parallel between C_2_ and R_3_ (Figure 3a, Figure 4a and Figure 5c,e) corresponding to an interfacial electrolyte/electrode EDLC. A diffusive element is also present and represented by Wd or Ma; the diffusion may take place from the EDLC at the interface and the diffusive element will be in series with the resistor representing the resistance of the EDLC. However, when the EDLC is either not representative or not existing, the diffusive element is independent and associated in series to the main equivalent circuit.

The PEIS analyses obtained immediately after cell assembly and after the CVs at 0.1, 25 and 50 mV·s^−1^, show an additional semicircle (Figure 3a, Figure 4a and Figure 5c,e) corresponding to R_3_ in parallel with C_2_. This semicircle may represent the resistance to the ionic movement in an EDLC at one of the interfaces of the Cu/Na_2.99_Ba_0.005_ClO/Zn cell, which formed spontaneously to equalize the electrochemical potentials of the electrolyte and the CCs. Table 2 presents the corresponding resistances, which demonstrate a downward trend in the total resistance up to the fourth CV, indicative of enhanced ionic conductivity upon cycling.

In the context of CV analysis, it is possible to establish charge transfer kinetics, and identify multistage reaction mechanisms. Furthermore, these analyses enable the determination of the charge storage capacity, along with the identification of capacitive and pseudocapacitive behavior. Additionally, through cycle scan, it is possible to analyze diffusion limitations in electrochemical reactions, as well as monitor the stability of CCs and electrolytes: material degradation, and/or the durability of electrochemical devices.

Here, the working range on the CVs was set at between −0.4 and 1.5, 2.0, or 4.0 V. The behavior shows redox peaks at 1.2 V, while charging, and at 1.0 V and 0.65 V, while discharging, due to tunneling of electrons at 0.1 mV·s^−1^. The OCV is 1.1 ∈ ]1.0, 1.2[ (V). The difference in chemical potentials of the CCs that substitute the traditional electrodes is $OCV=\left[\mu_{Zn\left(-\right)}-\mu_{Cu\left(+\right)}\right]/e\approx 1.1$ V. While charging above 1.1 V, Zn is reduced and Cu oxidized and while discharging below 1.1 V, Cu is reduced and Zn oxidized.

Over the course of the cycles, there is a decrease in hysteresis. This phenomenon is seen more clearly in the experiments with lower scan rates, mainly 0.1 and 1 mV·s^−1^, as expected, as the lower the rate, the closer to equilibrium. It is important to note that the ferroelectric behavior of the electrolyte is detected in these analyses because the first measured point shows high polarization; the cell is then polarized in another direction with the application of the electric field. Spontaneous polarization is likely essential while enabling charge transfer between current CCs and perhaps electrodes.

**Figure 6:**
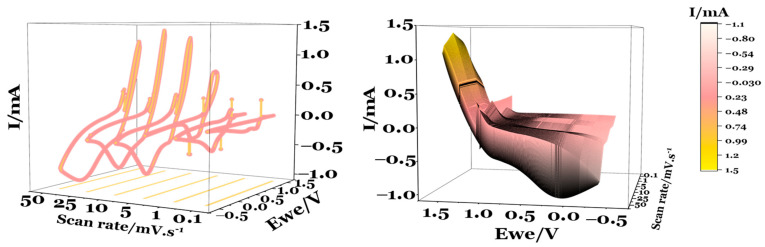
Electrochemical performance of a Cu/Na_2.99_Ba_0.005_ClO composite in cellulose/Zn pouch cell (PC I): 3D surface maps comparing the CVs at different scan rates.

**Figure 7:**
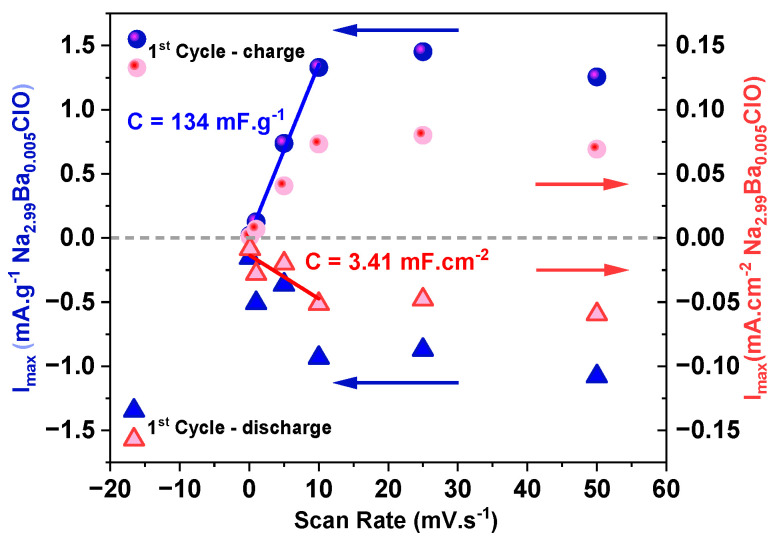
Maximum current obtained with a Cu/Na_2.99_Ba_0.005_ClO in cellulose/Zn pouch cell (PC I) for the first cycle upon charge and discharge at 0.1, 1, 5, 10, 25, and 50 mV·s^−1^.

To clearly visualize and directly identify the optimal working window, the experimental results from the CV were compiled into a 3D graph, incorporating the three key variables: (1) current (I, mA), (2) electrode potential (Ewe, V), and (3) scan rate (mV·s^−1^) (Figure 6). There is a significant variation in response to different scan rates, with higher currents being achieved at the same potential at faster scan rates, as expected. However, for 10 ≤ scan rates (mV·s^−1^) ≤ 50, the maximum current achieved is approximately constant at 1.3–1.4 mA (Figure 7).

The focus of these cells is the ferroelectric solid electrolyte, rich in Na^+^ ions, with a theoretical specific capacity of 663 mAh·g^−1^.

A resistor with 553 kΩ was connected in parallel with the cell and monitored using a battery tester, in voltmeter mode. This setup shows the cell’s voltage under a constant resistance (Figure 5f). Initially, the cell’s open-circuit voltage (OCV) was approximately 1.06 V, corresponding to Zn and Cu chemical potential difference. Surprisingly, over the course of 3000 h, the voltage gradually increased to 1.13 V, even if the cell continued to supply energy to the material resistor. This voltage surge is closely linked to the intrinsic properties of ferroelectric materials, particularly the orientation of internal electric dipoles (Figure 2b). These findings indicate the cells have self-charging capability.

The quantitative data for the cell in Figure 5f, while connected to a constant resistance (CR) of 553 kΩ, is shown in Table 3. The CR corresponds to a current of approximately 0.002 mA (2 μA). In discharge mode, the cell showed a capacity of approximately 6.0 mAh, corresponding to 5.9 mAh·g^−1^_electrolyte_, which equals a deposition thickness of metallic sodium on the negative CC (zinc) of approximately 3.0 μm, assuming homogeneous deposition and the existence of electronic positive feedback through the surface of the electrolyte, as demonstrated hereafter. The electrochemical discharge was forcefully stopped; therefore, the capacity of the cell is not the maximum for the corresponding resistor.

Figure 4 shows the maximum peak currents observed during the CV for both charge and discharge steps. At lower scan rates (0.1, 1, 5, 10 mV·s^−1^), the current rises linearly. The calculated capacitance is 134 mF·g^−1^ and 3.41 mF·cm^−2^. However, at higher scan rates (25 and 50 mV·s^−1^), the current values show minimal variation. This suggests that beyond 10–25 mV·s^−1^, the cell may struggle to respond to further increases in scan rate. In other words, the charges do not resonate with frequencies imposed by scan rates above 10–25 mV·s^−1^.

**Figure 8:**
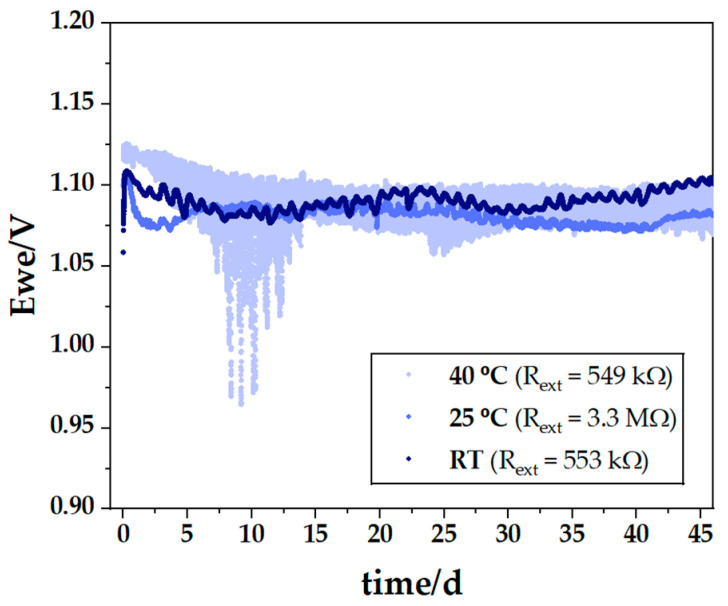
Comparison of the discharge behavior of Cu/Na_2.99_Ba_0.005_ClO composite in cellulose/Zn pouch cells with similar and different external resistors and different temperatures: 40 °C (PC II), 25 °C (PC III) and room temperature (PC I). Note: the electrochemical discharges were forcefully stopped.

Cells assembled as the cell in Figure 3, Figure 4, Figure 5 and Figure 6, were tested at different temperatures (Figure 8). One cell (PC II in Table 1) was immersed in a sand bath at 40 °C and connected to a 549 kΩ resistor, another (PC III in Table 1) was maintained at 25 °C and connected to a 3.3 MΩ resistor, and the last (PC I in Table 1) was left at room temperature and connected to a 553 kΩ resistor, where it experienced natural temperature fluctuations in the laboratory—cooler at night and warmer during the day. The results indicated that the cell maintained at room temperature and connected to a 553 kΩ resistor gave the most favorable performance, with the smallest oscillation range and a consistent trend toward higher potential difference (Figure 8). The comparison between the cell at 25 °C connected to a 3.3 MΩ resistor and the cell at room temperature connected to a 553 kΩ resistor showed no advantage in keeping the cell at OCV (or 3.3 MΩ) and at a fixed temperature of 25 °C.

The present cells benefit from being stored in a closed circuit with a small relative current. The temperature variations may enhance the polarization of the cell due to the pyroelectric character of the ferroelectric electrolyte. At 40 °C, the oscillations with higher amplitude between day 5 and 15, are likely indicative of the difficulty in plating sodium on Zn while discharging. As demonstrated hereafter, plating does not happen while discharging the cell at 40 °C.

It was anticipated that the cell’s performance at 40 °C would be superior to that at room temperature, based on a previous study that identified the electrolyte’s glass transition occurring between 46 and 48 °C [60] and, therefore, the rise in ionic conductivity. However, the chemical and electrochemical stability of the CCs in contact with the electrolyte are equally crucial and must be considered. The results shown in Figure 8 suggest that the CCs might be experiencing thermodynamic instability, leading to undesirable side reactions that cause chemical corrosion. Alternatively, the additional kinetic energy, enhancing ionic conductivity may disrupt the electronic feedback conduction through the surface of the electrolyte Na_2.99_Ba_0.005_ClO.

#### 2.1.3. SEM and EDX Analysis

**Figure 9:**
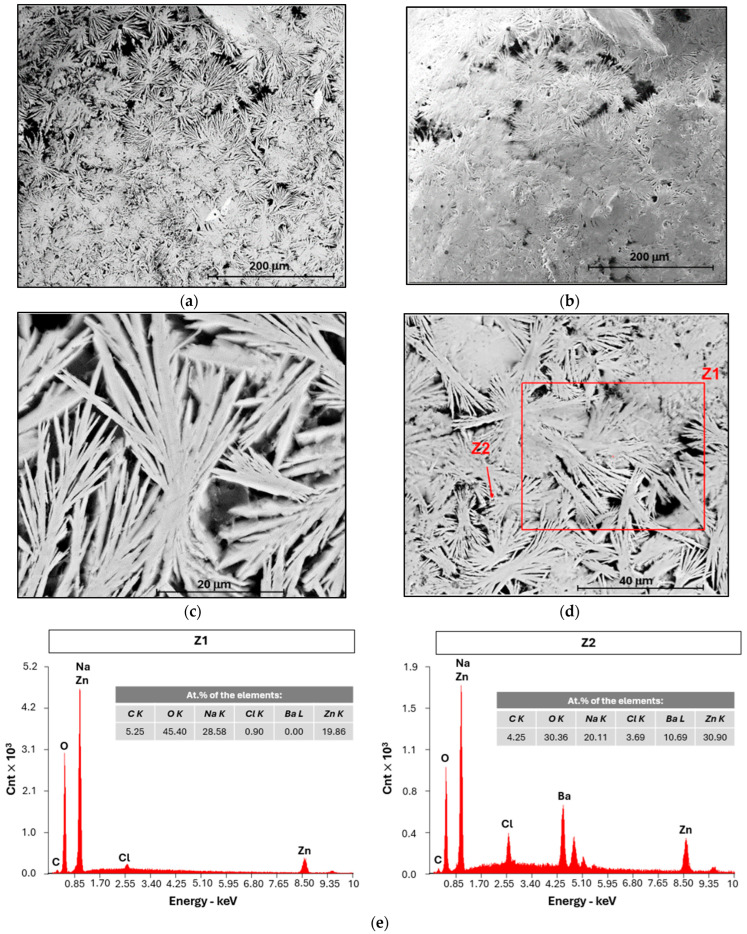
Characterization of the zinc foil of PC IV by SEM/EDX acquired using (**a**) back-scattered electron BSED detector with a magnification of 500×; (**b**) secondary electron SE detector with a magnification of 500×; (**c**) back-scattered electron BSED detector with a magnification of 5000×; (**d**) magnification 2500× with identification of the zones analyzed with EDX; (**e**) EDX of the red zones marked in Figure 9d, with an electron beam energy of 15 keV.

SEM/EDX is a semi-quantitative technique that provides complementary information to other analytical methods. After allowing a cell (PC IV in Table 1) to discharge for approximately 1436 h (~60 days) while connected to a 982 kΩ resistor, it showed a potential difference of 1.10 V corresponding to a capacity of 1.6 mAh (2.7 mAh·g^−1^_electrolyte_). The cell was then opened in a glove box, and two samples from the negative CC (zinc foil) were prepared and analyzed using SEM/EDX (Figure 9).

The SEM images reveal that the distribution of sodium on the surface of the CC is not uniform, resulting in a branched structure that expands in the plane of the Zn CC, as predicted in the simulations of Figure 2. When the branches meet, the Na layer becomes compacted. Figure 9a,b illustrate this heterogeneity at a lower magnification, while Figure 9c,d show it at higher magnification. By analyzing the marked zones in Figure 9d, the presence of the elements of interest can be identified (Figure 9e). In the larger area (Z1), sodium metal is clearly detected, whereas chlorine shows a very low atomic percentage, very deviated from the electrolyte and barium is undetectable due to its low concentration. Barium could be detected in more localized areas, such as the Z2 zone dispersed throughout the sample.

The clear deposition of metallic sodium, on the negative CC of a cell that was not charged by external electrical work, is the unequivocal proof that an internal feedback circuit of surface electrons in the Na_2.99_Ba_0.005_ClO ferroelectric electrolyte, moving from the positive (Cu) to the negative (Zn) CC, reduces the Na^+^ in the electrolyte at the interface with Zn in accordance with the equation ${Na}^{+}+e^{-}↔Na$ resulting in the increase in the chemical potential bias and, therefore, the cell voltage. The Na metal is instantaneously oxidized.

**Figure 10:**
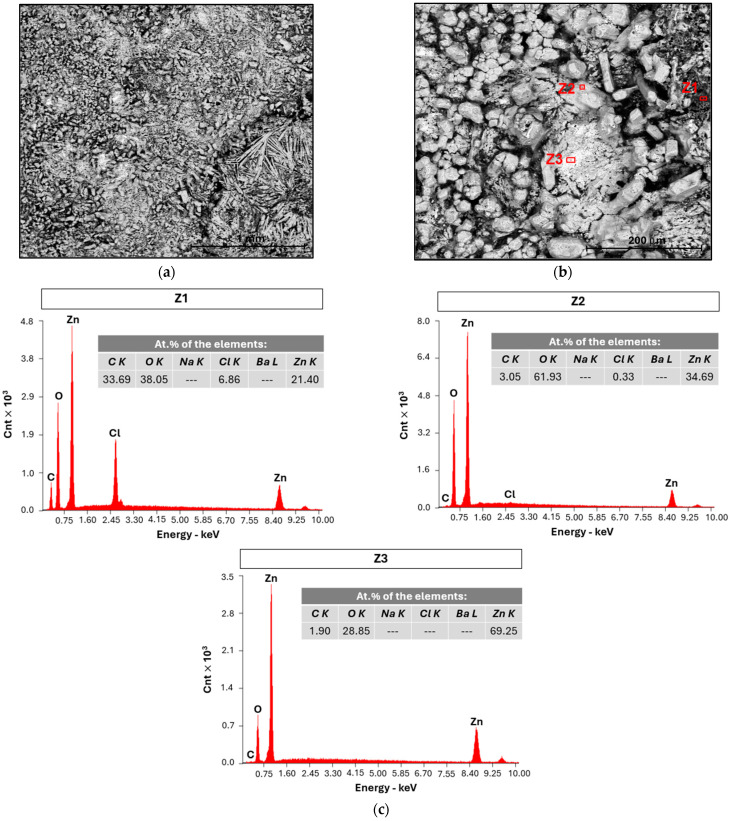
Characterization of the zinc foil of PC II by SEM/EDX acquired using (**a**) back-scattered electron BSED detector with a magnification of 100×; (**b**) magnification 500× with identification of the zones analyzed with EDX; (**c**) EDX of the red zones marked in Figure 10b, with an electron beam energy of 15 keV.

The zinc sheet of PC II (Table 1) that was analyzed at 40 °C, shown in Figure 10, helps clarify the discharge results in Figure 8. It can be verified that there is an absence of Na, which indicates that the oxidation of the Zn foil overcomes the nucleation of Na, as further confirmed by EDX analysis (Figure 10c).

### 2.2. Performance of Cu/Carbon Felt/Na_2.99_Ba_0.005_ClO Composite in Cellulose/Zn Pouch Cell

To enhance the performance of the presented cells, a layer of carbon felt was added to the positive side, adjacent to the copper sheet, fabricating a cell with the same configuration as described in the previous section.

#### Electrochemical Cycling

**Figure 11:**
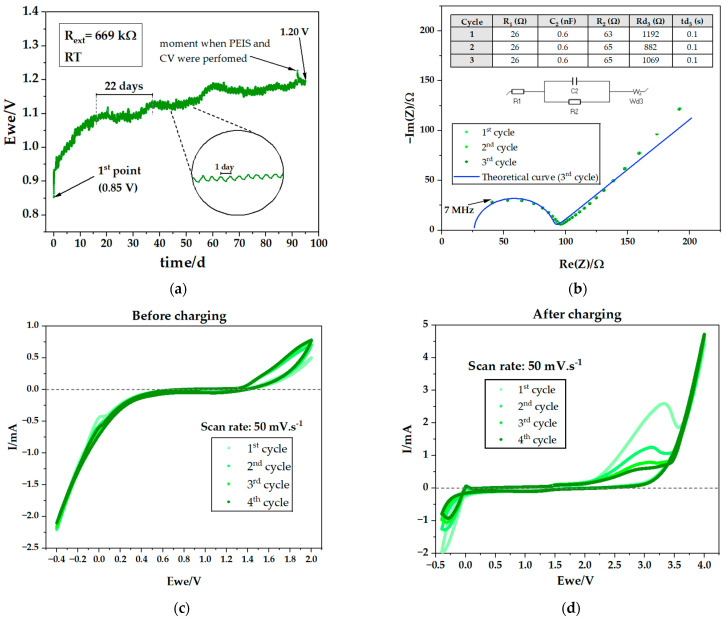
Electrochemical performance of a Cu/carbon felt/Na_2.99_Ba_0.005_ClO composite in cellulose/Zn pouch cell (PC V); (**a**) discharge analysis with an external resistance of 669 kΩ associated in parallel at room temperature; (**b**) PEIS analysis performed after 2206 h (approx. 92 days) discharge in Figure 5a; (**c**) CV analysis at 50 mV·s^−1^ performed immediately after PEIS; (**d**) CV analysis at 50 mV·s^−1^ performed after charging the cell to 3 V for 23 h at room temperature.

The initial OCV of the cell (PC V in Table 1) was recorded at 0.85 V, considerably lower than that of the cell without the carbon felt (CF). However, over time, the potential difference in the cell system connected to an external resistance of 669 kΩ during the discharge stage steadily increased. After 2277 h, it reached 1.20 V, surpassing the voltage of the cell without CF (Figure 11a). The characteristic oscillations attributed to the ferroelectric electrolyte are also visible. Quantitative analysis in Table 4 indicates that the current in this cell is consistent with the previous one, although exhibiting a lower discharge capacity as the operating time was lower and, therefore, corresponding to a metallic sodium deposition thickness on Zn of approximately 2.3 μm (2.5 mAh·g^−1^). As in the cells absent of carbon, the electronic feedback was established.

Following approximately 2207 h of cell discharge, PEIS and CV were performed to gain deeper insights into the cell’s behavior, as demonstrated in Figure 11a. The PEIS are shown in Figure 11b, while Figure 11c shows the CV performed at 50 mV·s^−1^. PEIS clearly show the three cycles overlap, indicating the cell is highly stable. In addition, the low resistances indicate the electrochemical system is operating efficiently with minimal losses. The PEIS supports the conclusion that the cell’s electrolyte is facilitating fast ionic movement, which is critical to the efficient performance of the device. PEIS is also typically performed to assess cell integrity. In this context, the low resistance further indicates the cell remains in good condition, with no significant degradation. Examining the CVs performed between −0.4 V and 2 V (Figure 11c), it is observed that the reduction in the Zn and C/Cu oxidation starts at 1.3 V.

The CV displayed in Figure 11d was the result of a preceding 23 h conventional charge at 3 V. In this CV, the voltage range was extended to 4 V, revealing an exponential increase in the higher voltage values. Furthermore, a reduction in hysteresis over cycles is evident, particularly within the 2 to 3.3 V range. This CV shows the formation of Na metal at negative voltages, where the collector at higher chemical potential is C/Cu; the absolute current is I = −2.0 mA (first cycle) increasing to zero and plateauing from 0.2 to 2.3 V to then show redox activity up to 4 V. It is worth highlighting the OCV of the cell containing Na metal plated on Zn is $\left[\mu \left(Na\right)-\mu \left(C\right)\right]/e=3.2$ V while the OCV of the Zn//Cu cell is $\left[\mu \left(Zn\right)-\mu \left(C\right)\right]/e=1.3$ V, which indicates that the redox activity above approximately 1.3 V corresponds to a cell different from Zn//C. In this case, the redox peak at ~3.3 V is indicative of the presence of sodium on the negative CC. The latter discussion is reflected in the differences between the CVs in Figure 5d, where the electrochemical activity is a mirror of the ferroelectric activity of Na_2.99_Ba_0.005_ClO, and Figure 11c,d, where the plated Na metal reinforces the redox activity at negative voltages and above 1.3 V.

**Figure 12:**
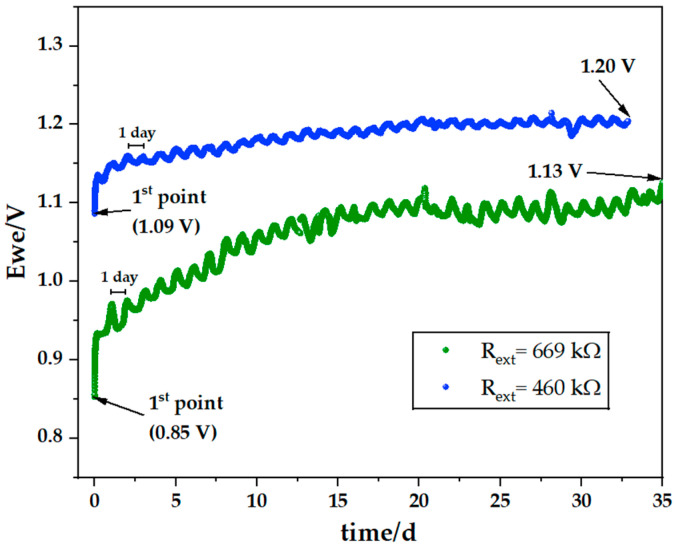
Comparison between the discharge behavior of two pouch cells with a configuration Cu/carbon felt/Na_2.99_Ba_0.005_ClO composite in cellulose/Zn: (green) cell connected to a 669 kΩ external resistor (PC V) and (blue) cell connected to a 460 kΩ external resistor (PC VI).

Figure 12 shows the discharge of two cells with similar configurations but connected to different resistors. The cell connected to a lower resistance (PC VI in Table 1) showed a higher initial voltage 1.09 vs. 0.85 V and kept the voltage above the cell with a higher resistance. This is not expected, but points to the existence of an optimal current.

In contrast, the cell connected to the higher resistance (PC V in Table 1) showed a more pronounced evolution with a higher increment of the potential difference over 789 h (0.25 vs. 0.11 V), indicating superior self-charging. Table 4 presents a detailed quantitative analysis of the results for both cells.

## 3. Materials and Methods

### 3.1. Materials

For the electrode-less pouch cells fabricated in this study, copper sheets with 99.9% metal purity and dimensions of (0.127 × 35 × 50) mm^3^ were used as the positive CC, while zinc sheets with 99.98% metal purity and (0.25 × 35 × 50) mm^3^ were used as the negative CC. The electrolyte employed was Na_2.99_Ba_0.005_ClO, a glassy ferroelectric electrolyte synthesized through water solvation, as described by Braga et al. [14], using NaCl (purity 99.0%), NaOH (anhydrous, purity 99%), and Ba(OH)_2_ (anhydrous, purity 94–98%) as precursors. Cellulose sheets with dimensions of (0.07 × 40 × 55) mm^3^ were then impregnated with the Na_2.99_Ba_0.005_ClO, to serve as the separator. To vacuum seal the pouch cells, an aluminum-laminated film with (70 × 140) mm^2^ folded in half by the most significant dimension, was used. Later, carbon felt with a purity of 99.0% and (1.59 × 35 × 50) mm^3^ was introduced on the positive side of the pouch cells.

**Figure 13:**
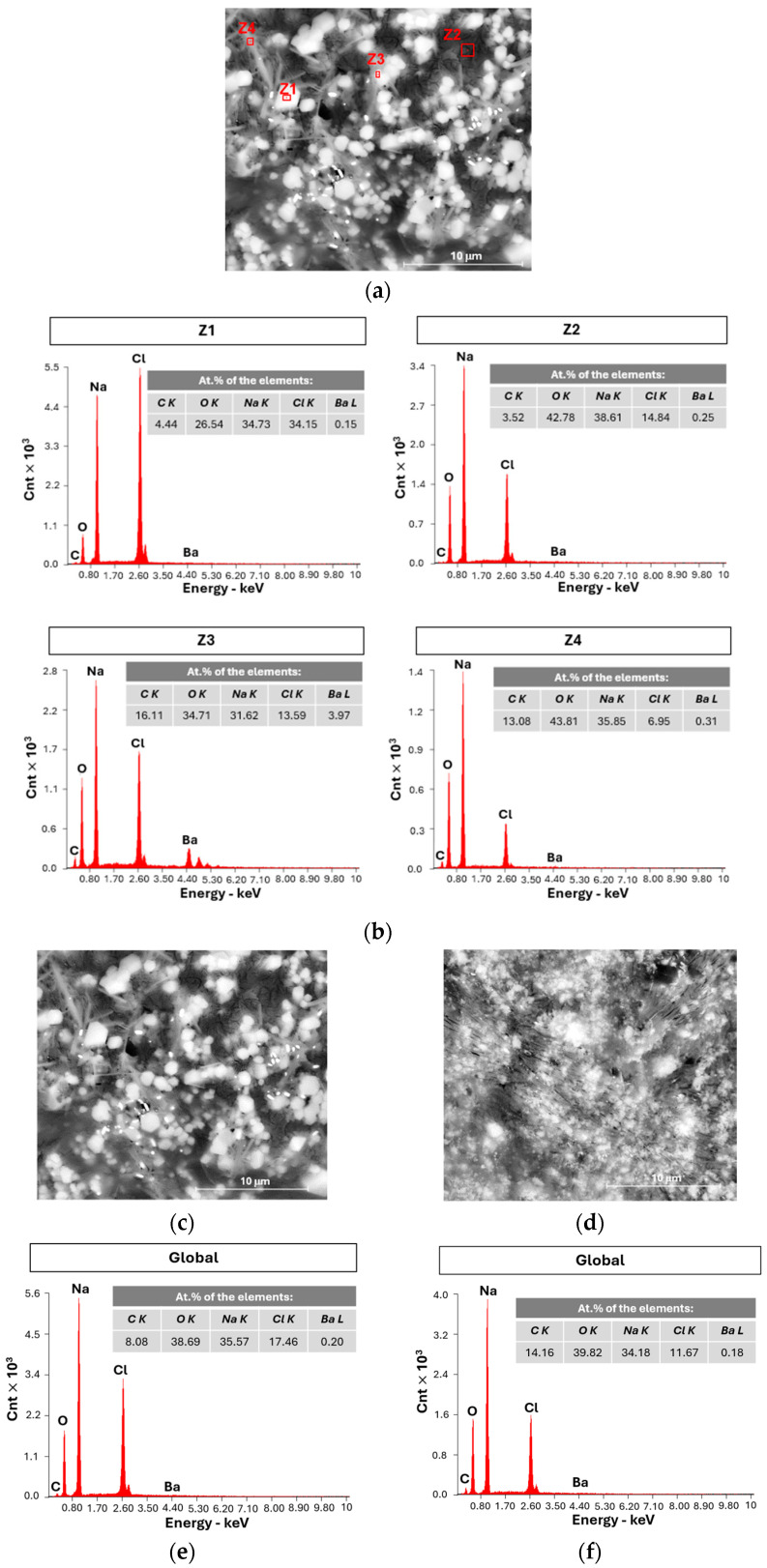
Characterization by SEM/EDX images acquired with the BSED detector (**a**) powder Na_2.99_Ba_0.005_ClO with 10,000× magnification, with identification of the zones analyzed by EDX; (**b**) EDX results of the red zones marked in Figure 13a, with an electron beam energy of 15 keV; (**c**) powder Na_2.99_Ba_0.005_ClO, magnified 10,000×; (**d**) powder mixture comprising 80% Na_2.99_Ba_0.005_ClO and 20% PVA_C_, magnified 10,000×; (**e**) EDX results for the global analysis correspondent to Figure 13c, with an electron beam energy of 15 keV; (**f**) EDX results for the global analysis correspondent to Figure 13d, with an electron beam energy of 15 keV.

The electrolyte powder Na_2.99_Ba_0.005_ClO and a mixture of 80% Na_2.99_Ba_0.005_ClO with 20% PVA_C_ (powder) were analyzed using SEM/EDX, as shown in Figure 13. These images demonstrate that the mixture is more homogeneous (Figure 13d) than the plain electrolyte in Figure 13c. The PVA_C_, which acts as a binder, joins the electrolyte particles and creates a less hygroscopic composite than the plain Na_2.99_Ba_0.005_ClO. Consequently, it enhances the mechanical strength of the separator. The samples containing pure electrolyte show multiple whitish areas (crystals) that correspond to NaCl (Z1 e Z3, Figure 13b). These crystals are much less frequent in the mixture of electrolyte and PVA_C_, which suggests the mixture does not react with water as rapidly when exposed to a moist environment. When Na_3_BaClO absorbs moisture, NaCl and 2NaOH are formed.

The semi-quantitative EDX analyses allow for both global and localized analysis of the samples, enabling the identification of elements and their specific distribution within different regions (Figure 13b,e,f). In both samples, the elements are found to be evenly distributed, including barium, which, despite its lower concentration, is consistently present across all analyzed zones. The global composition shows x(Na/2.99) = 11.43, which is 98% of the x(Cl), as expected, and x(Na/2.99) × 0.005 = 0.057, which is ten times the concentration of Ba in Na_2.99_Ba_0.005_ClO.

### 3.2. Methods

#### 3.2.1. Ab Initio Simulations of the Cu/Na_3_ClO/Zn Cell

A surface can be considered a specific type of heterogeneous interface, which delineates two distinct phases in equilibrium. In the case of a surface, one of these phases would be vapor. Consequently, any discussion regarding surfaces or interfaces generally applies to both. In an interface or surface system in thermodynamical equilibrium, the chemical potential of each component must be identical in every phase (equilibrium condition). Following this principle, this study utilized the interfaces module as implemented in MedeA 3.7 to simulate the Cu/Na_3_ClO and Na_3_ClO/Zn interfaces. The software uses the latter equilibrium common approach in ab initio thermodynamics to examine the stability of surfaces and interfaces. Density functional theory (DFT) methods, implemented through the VASP 6.3.2 software [61] were conducted on Cu/Na_3_ClO/Zn to optimize the final heterojunction with a total of 87 atoms including (Cu)_52_, (Zn)_20_, and (Na_3_ClO)_3_. The GGA-PBE exchange–correlation functional was utilized for describing the interactions. A plane-wave energy cutoff of 400 eV and a k-point spacing of 0.2 Å^−1^ were used, generating a 7 × 2 × 2 mesh.

A >10 Å vacuum layer was added to the Cu/Zn interface to create two surfaces. The electron localization functions (ELF) were computed for the heterojunction and plotted for [100] Miller indices for different x-coordinates. These simulations utilized tools within VASP in the MedeA 3.7 software.

#### 3.2.2. Preparation of Materials

##### The Current Collectors

Given the cells are electrode-less, optimal sodium nucleation on the surface of both CCs is crucial for enhancing cell performance. To promote this nucleation, it is essential to introduce some texture on their surface; it was achieved manually using a P320 sandpaper.

##### The Separator

The separator preparation method followed the procedure outlined by Braga’s research group [14,62]. After synthesis and thorough drying, the electrolyte was finely ground into powder using a FRITSCH Planetary Mono Mill PULVERISETTE 6, FRITSCH Milling & Sizing, Inc., Pittsboro, NC, USA in an airtight agate container with five 20 mm diameter balls, operating at 300 rpm for 40 min. Subsequently, the electrolyte powder was mixed with absolute ethanol, followed by the addition of thermoplastic polyvinyl acetate (PVA_C_), commonly known as white glue, in a weight ratio of 80% Na_2.99_Ba_0.005_ClO to 20% PVA_C_. Once a homogeneous mixture was achieved, it was impregnated onto cellulose sheets, which were then placed inside a vacuum oven at 70 °C for at least 24 h until completely dried.

#### 3.2.3. Pouch Cell Manufacturing

The entire assembly process of these cells can be conducted outside of a glove box, due to the absence of hazardous or flammable materials. However, a swift assembly is crucial to prevent the electrolyte from absorbing moisture.

**Figure 14:**
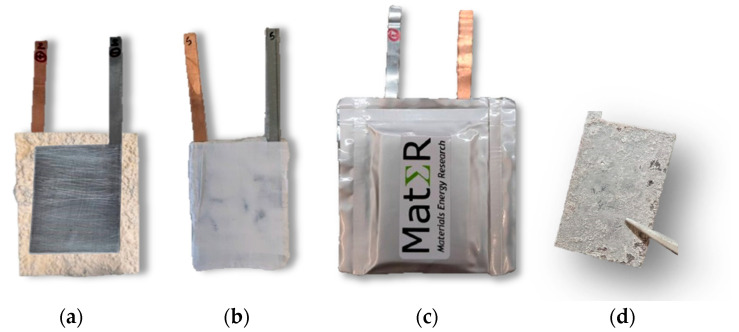
Schematic of various stages of a pouch cell study: (**a**) pouch cell assembled; (**b**) pouch cell involved in Teflon tape; (**c**) pouch cell vacuum-sealed inside an aluminum-laminated film; (**d**) postmortez zinc foil used in SEM/EDX prepared inside the glove box.

As detailed in Figure 14a–c, to assemble a cell, the separator must be positioned between the two CCs, ensuring they do not contact each other to avoid potential short circuits. Teflon tape is then applied to secure all the components in place. Finally, the cells undergo vacuum-sealing with a pilot line. Initially, an aluminum-laminated film pouch was formed using a pouch case-forming machine. Afterward, the top and one of the sides of the film were heat-sealed using a top-and-side heat-sealing machine. Lastly, a vacuum pre-sealing machine evacuated all the air from inside the pouch before heat-sealing the remaining side.

#### 3.2.4. Electrochemical Measurements

PEIS is a non-invasive technique that employs a potentiostat to apply a small amplitude AC signal superimposed on a fixed DC potential, allowing for the measurement of impedance response across a spectrum of frequencies. This technique yields valuable insights into the kinetics of electrochemical processes occurring at the interfaces within the cell [63,64,65]. For each cell, a sequence of three cycles over a frequency range of 1 MHz to 0.1 Hz with an AC signal amplitude of 10 mV were conducted.

CV is a versatile electrochemical technique characterized by its ability to probe the intricate redox processes within an electrochemical cell. This technique involves the linear sweeping of the voltage at a constant scan rate between two predefined limits while simultaneously recording the resultant current. By analyzing the CV curves, we gain critical insights into the oxidation and reduction mechanisms, reaction kinetics, and electrochemical behavior of the cell components [66,67].

The voltage varied systematically from −0.4 V to 2.0 V, and various scan rates—0.1, 1, 5, 10, 25, and 50 mV·s^−1^—were used to evaluate the influence of the scan rate on the electrochemical response.

For the pouch cells without electrodes, the discharge cycles were evaluated by connecting them in parallel to different external resistors. These configurations were connected and analyzed in two distinct devices: the Neware CT-4008Tn 5V/20mA battery tester, Neware Technology, Shenzhen, China and the BioLogic VMP-300 potentiostat/galvanostat/impedance spectroscope, BioLogic Corporation, Seyssinet-Pariset, France.

The data collected in the laboratory were transferred to OriginPro 2020^®^, OriginLab Corporation, Northampton, MA, USA.

#### 3.2.5. SEM/EDX Analysis and Sample Preparation

Scanning electron microscopy with energy-dispersive X-ray spectroscopy (SEM/EDX) was also performed to analyze the surfaces of both the positive and negative CCs. SEM/EDX is a widely employed technique for determining the chemical composition of samples. This method works by directing an electron beam (with an energy of 15 keV) onto the sample, exciting the atoms within it. This excitation causes the sample to emit X-rays, which are then detected and analyzed to identify and quantify the elements present. SEM/EDX can identify all elements except hydrogen H, helium He, and lithium Li [68]. The SEM/EDX analysis was performed using a high-resolution (Schottky) environmental scanning electron microscope with X-ray microanalysis and electron backscattered diffraction analysis: FEI Quanta 400 FEG ESEM/EDAX Genesis X4M, FEI Company, Hillsboro, OR, USA.

Before preparing the samples, a PEIS and a CV were performed on the cell. The sample preparations were conducted within a controlled atmosphere glove box, maintaining oxygen (O_2_) and water (H_2_O) levels below 1 ppm. The cells were opened (see zinc foil, Figure 14d), and the surfaces were marked and cut accordingly into samples of approximately 1 cm^2^ in size. These samples were kept in a vacuum until ready for analysis. Two samples were collected from each CC. Two samples of the electrolyte powder, one with and one without PVA_C_ were also analyzed (see Section 3.1). It was not necessary to apply a coating to any of the samples, as they were already conductive.

## 4. Conclusions

This study discusses sustainable solid-state batteries that operate without traditional electrodes. These cells consist of CCs made from copper and zinc foils and a ferroelectric electrolyte composed of Na_2.99_Ba_0.005_ClO. This innovative electrolyte is a key contributor to the batteries’ exceptional Na-plating and self-charge performance.

The unitary pouch cell, consisting solely of a Cu/Na_2.99_Ba_0.005_ClO composite in cellulose/Zn, demonstrated the achievement of an OCV of approximately 1.10 V, after assembly. When set to discharge, despite operating with a very low current of 0.002 mA, the cell exhibited a potential difference higher than the initial (1.13 V) after approximately 120 days, resulting in a capacity of 5.9 mAh·g^−1^_electrolyte_. Notably, this cell lacks traditional electrodes, so its capacity is entirely derived from the electrolyte, which is rich in Na^+^ ions.

A thin sheet of carbon felt was then added adjacent to the copper sheet, resulting in an OCV of approximately 0.85 V after assembly. However, with a very low discharge current, similar to the one of the cell without carbon felt, a potential difference of 1.20 V was achieved after 95 days, corresponding to a capacity of 2.5 mAh·g^−1^_electrolyte_. The capacity should have been indicative of plating or Faradaic reactions on the C/Cu collectors as the cell was set to discharge. However, the calculated thickness of sodium deposited on a CC is very similar to the thickness of sodium plated on zinc determined by SEM/EDX, indicating a positive feedback current through the surface of the electrolyte that reduces the Na^+^ in the interface with the Zn.

To provide additional evidence to support the experimental findings, ab initio simulations of the Cu/Na_3_ClO/Zn heterojunction were conducted. These simulations demonstrated the formation of NaO^−^ dipoles and the deposition of sodium on the surface of the zinc CC.

The results presented in this article highlight the significant potential of the Na^+^-rich ferroelectric electrolyte, paving the way to developing sustainable and environmentally friendly batteries. Moving forward, it will be important to design batteries with compatible electrodes that enhance the performance of the electrolyte, which serves both as a separator and a source of mobile Na^+^ ions. The challenges mostly lie in increasing the output power. One of the possible approaches is to use a conventional cathode to increase the bias between the Zn (negative electrode/CC) and the positive electrode/CC. The future of battery technology should be driven by sustainability.

## Figures and Tables

**Table 1:** Identification of the pouch cells and corresponding weights of separator components (Na_2.99_Ba_0.005_ClO electrolyte, PVA_C_, and cellulose).

# Cell	m_separator_ (g)	m_cellulose_ (g)	$m_{{Na}_{2.99}{Ba}_{0.005}ClO+PVAc}$ for (4.0 × 5.5) cm^2^ (g)	$m_{{Na}_{2.99}{Ba}_{0.005}ClO}$ for (4.0 × 5.5) cm^2^ (g)	$m_{{Na}_{2.99}{Ba}_{0.005}ClO}$ for (3.5 × 5.0) cm^2^ (g)
PC I	1.630	0.044	1.586	1.269	1.009
PC II	1.250	0.044	1.206	0.965	0.768
PC III	1.895	0.045	1.850	1.480	1.177
PC IV	0.978	0.046	0.932	0.746	0.593
PC V	2.916	0.046	2.870	2.296	1.826
PC VI	1.584	0.046	1.538	1.230	0.978

**Table 2:** Summary of the resistances obtained by PEIS with a Cu/Na_2.99_Ba_0.005_ClO composite in cellulose/Zn pouch cell (PC I).

	1st Cycle			2nd Cycle			3rd Cycle		
	R_1_ (Ω)	R_2_ (Ω)	R_3_ (Ω)	R_1_ (Ω)	R_2_ (Ω)	R_3_ (Ω)	R_1_ (Ω)	R_2_ (Ω)	R_3_ (Ω)
After assembly	86	68	39	79	80	39	91	71	38
	Σ = 193			Σ = 198			Σ = 200		
After 1st CV	33	109	259	36	222	140	39	241	120
	Σ = 401			Σ = 398			Σ = 400		
After 2nd CV	27	147	-	48	153	-	45	160	-
	Σ = 174			Σ = 201			Σ = 205		
After 3rd CV	20	178	-	36	164	-	55	139	-
	Σ = 198			Σ = 200			Σ = 194		
After 4th CV	31	103	-	63	103	-	74	98	-
	Σ = 134			Σ = 166			Σ = 172		
After 5th CV	69	144	142	59	62	146	62	40	131
	Σ = 355			Σ = 267			Σ = 233		
After 6th CV	64	153	42	55	164	52	59	166	47
	Σ = 259			Σ = 271			Σ = 272		

**Table 3:** Cu/Na_2.99_Ba_0.005_ClO in cellulose/Zn pouch cell (PC I) capacity while set to discharge at constant resistance at room temperature. Surface area A = 17.5 cm^2^. Note: the electrochemical discharge was forcefully stopped.

Open Circuit Voltage OCV (V)	External Resistor R_ext_ (kΩ)	Discharge Time Time (h)	Maximum Discharge Potential E_we_ (V)	Discharge Current I_Rext_ (mA)	Discharge Capacity Q_Rext_ (mAh)	Thickness Na on Zn(−) d_Na_ (μm)
1.06	553	3003	1.13	0.002	5.996	3.0

**Table 4:** Cu/carbon felt /Na_2.99_Ba_0.005_ClO in cellulose/Zn pouch cell (PC V) capacities while the cell is set to discharge at constant external resistance at room temperature. Surface area A = 17.5 cm^2^.

Open Circuit Voltage OCV (V)	External Resistor R_ext_ (kΩ)	Discharge Time Time (h)	Last Discharge Potential E_we_ (V)	Maximum Discharge Current I_Rext_ (mA)	Discharge Capacity Q_Rext_ (mAh)	Thickness Na in Zn d_Na_ (μm)
0.85	669	2277	1.20	0.002	4.554	2.3
1.09	460	789	1.10	0.002	1.578	0.8

## Data Availability

Data are available on reasonable request.

## References

[1] Akpan J., Olanrewaju O. (2023). Sustainable Energy Development: History and Recent Advances. Energies.

[2] Batra G. (2023). Renewable Energy Economics: Achieving Harmony between Environmental Protection and Economic Goals. Soc. Sci. Chron..

[3] Hamdan A., Daudu C.D., Fabuyide A., Etukudoh E.A., Sonko S. (2024). Next-Generation Batteries and US Energy Storage: A Comprehensive Review: Scrutinizing Advancements in Battery Technology, Their Role in Renewable Energy, and Grid Stability. World J. Adv. Res. Rev..

[4] Kebede A.A., Kalogiannis T., Van Mierlo J., Berecibar M. (2022). A Comprehensive Review of Stationary Energy Storage Devices for Large Scale Renewable Energy Sources Grid Integration. Renew. Sustain. Energy Rev..

[5] Zhu Z., Jiang T., Ali M., Meng Y., Jin Y., Cui Y., Chen W. (2022). Rechargeable Batteries for Grid Scale Energy Storage. Chem. Rev..

[6] Elalfy D.A., Gouda E., Kotb M.F., Bureš V., Sedhom B.E. (2024). Comprehensive Review of Energy Storage Systems Technologies, Objectives, Challenges, and Future Trends. Energy Strategy Rev..

[7] Wu D., Wu F. (2023). Toward Better Batteries: Solid-State Battery Roadmap 2035+. Etransportation.

[8] Thomas F., Mahdi L., Lemaire J., Santos D.M.F. (2024). Technological Advances and Market Developments of Solid-State Batteries: A Review. Materials.

[9] Schmaltz T., Hartmann F., Wicke T., Weymann L., Neef C., Janek J. (2023). A Roadmap for Solid-State Batteries. Adv. Energy Mater..

[10] Song Y., Sun X., Lou S., Sun F., Wang J. (2024). Alleviating Range Anxiety: Solid-State Batteries and Extreme Fast Charging. Prog. Mater. Sci..

[11] Shah R., Mittal V., Precilla A.M. (2024). Challenges and Advancements in All-Solid-State Battery Technology for Electric Vehicles. J.

[12] Kazyak E., García-Méndez R. (2024). Recent Progress and Challenges for Manufacturing and Operating Solid-State Batteries for Electric Vehicles. MRS Bull..

[13] Braga M.H., Oliveira J.E., Murchison A.J., Goodenough J.B. (2020). Performance of a Ferroelectric Glass Electrolyte in a Self-Charging Electrochemical Cell with Negative Capacitance and Resistance. Appl. Phys. Rev..

[14] Danzi F., Camanho P.P., Braga M.H. (2021). An All-Solid-State Coaxial Structural Battery Using Sodium-Based Electrolyte. Molecules.

[15] Wen J., Yu Y., Chen C. (2012). A Review on Lithium-Ion Batteries Safety Issues: Existing Problems and Possible Solutions. Mater. Express.

[16] Deberdt R., Le Billon P. (2021). Conflict Minerals and Battery Materials Supply Chains: A Mapping Review of Responsible Sourcing Initiatives. Extr. Ind. Soc..

[17] Wang W., Dutton S., Liu G., Manthiram A. (2022). Editorial for Special Issue on Abundant and Non-Toxic Materials for Batteries. APL Mater..

[18] Ryu H.-H., Sun H.H., Myung S.-T., Yoon C.S., Sun Y.-K. (2021). Reducing Cobalt from Lithium-Ion Batteries for the Electric Vehicle Era. Energy Environ. Sci..

[19] Lebrouhi B.E., Baghi S., Lamrani B., Schall E., Kousksou T. (2022). Critical Materials for Electrical Energy Storage: Li-Ion Batteries. J. Energy Storage.

[20] Bruno M., Fiore S. (2024). Review of Lithium-Ion Batteries’ Supply-Chain in Europe: Material Flow Analysis and Environmental Assessment. J. Environ. Manag..

[21] Lehtimäki H., Karhu M., Kotilainen J.M., Sairinen R., Jokilaakso A., Lassi U., Huttunen-Saarivirta E. (2024). Sustainability of the Use of Critical Raw Materials in Electric Vehicle Batteries: A Transdisciplinary Review. Environ. Chall..

[22] Nayak P.K., Yang L., Brehm W., Adelhelm P. (2018). From Lithium-ion to Sodium-ion Batteries: Advantages, Challenges, and Surprises. Angew. Chem. Int. Ed..

[23] Hafiz N.S.M., Singla G., Jha P.K. (2022). Next Generation Sodium-Ion Battery: A Replacement of Lithium. Mater. Today Proc..

[24] Eftekhari A., Kim D.-W. (2018). Sodium-Ion Batteries: New Opportunities beyond Energy Storage by Lithium. J. Power Sources.

[25] Yabuuchi N., Kubota K., Dahbi M., Komaba S. (2014). Research Development on Sodium-Ion Batteries. Chem. Rev..

[26] Ellis B.L., Nazar L.F. (2012). Sodium and Sodium-Ion Energy Storage Batteries. Curr. Opin. Solid. State Mater. Sci..

[27] Li F., Wei Z., Manthiram A., Feng Y., Ma J., Mai L. (2019). Sodium-Based Batteries: From Critical Materials to Battery Systems. J. Mater. Chem. A Mater..

[28] Liu Z., Lu Z., Guo S., Yang Q.-H., Zhou H. (2023). Toward High Performance Anodes for Sodium-Ion Batteries: From Hard Carbons to Anode-Free Systems. ACS Cent. Sci..

[29] Zhuang R., Zhang X., Qu C., Xu X., Yang J., Ye Q., Liu Z., Kaskel S., Xu F., Wang H. (2023). Fluorinated Porous Frameworks Enable Robust Anode-Less Sodium Metal Batteries. Sci. Adv..

[30] Guerreiro A.N., Costa I.B., Vale A.B., Braga M.H. (2023). Distinctive Electric Properties of Group 14 Oxides: SiO_2_, SiO, and SnO_2_. Int. J. Mol. Sci..

[31] Berlanga C., Monterrubio I., Armand M., Rojo T., Galceran M., Casas-Cabanas M. (2019). Cost-Effective Synthesis of Triphylite-NaFePO4 Cathode: A Zero-Waste Process. ACS Sustain. Chem. Eng..

[32] Hasa I., Mariyappan S., Saurel D., Adelhelm P., Koposov A.Y., Masquelier C., Croguennec L., Casas-Cabanas M. (2021). Challenges of Today for Na-Based Batteries of the Future: From Materials to Cell Metrics. J. Power Sources.

[33] Arnaiz M., Gómez-Cámer J.L., Gonzalo E., Drewett N.E., Ajuria J., Goikolea E., Galceran M., Rojo T. (2021). Exploring Na-Ion Technological Advances: Pathways from Energy to Power. Mater. Today Proc..

[34] Liu G., Yang J., Wu J., Peng Z., Yao X. (2024). Inorganic Sodium Solid Electrolytes: Structure Design, Interface Engineering and Application. Adv. Mater..

[35] Shi C., Takeuchi S., Alexander G.V., Hamann T., O’Neill J., Dura J.A., Wachsman E.D. (2023). High Sulfur Loading and Capacity Retention in Bilayer Garnet Sulfurized-Polyacrylonitrile/Lithium-Metal Batteries with Gel Polymer Electrolytes. Adv. Energy Mater..

[36] Braga M.H., Ferreira J.A., Stockhausen V., Oliveira J.E., El-Azab A. (2014). Novel Li_3_ClO Based Glasses with Superionic Properties for Lithium Batteries. J. Mater. Chem. A Mater..

[37] Chi X., Zhang Y., Hao F., Kmiec S., Dong H., Xu R., Zhao K., Ai Q., Terlier T., Wang L. (2022). An Electrochemically Stable Homogeneous Glassy Electrolyte Formed at Room Temperature for All-Solid-State Sodium Batteries. Nat. Commun..

[38] Eshetu G.G., Elia G.A., Armand M., Forsyth M., Komaba S., Rojo T., Passerini S. (2020). Electrolytes and Interphases in Sodium-based Rechargeable Batteries: Recent Advances and Perspectives. Adv. Energy Mater..

[39] Tao J., Chen Y., Bhardwaj A., Wen L., Li J., Kolosov O.V., Lin Y., Hong Z., Huang Z., Mathur S. (2022). Combating Li Metal Deposits in All-Solid-State Battery via the Piezoelectric and Ferroelectric Effects. Proc. Natl. Acad. Sci. USA.

[40] Zhu X., Xiong S., Zhu G., Chen D., Wang Z., Lei X., Liu L., Li C. (2024). Dielectric Properties and Excellent Energy Storage Density under Low Electric Fields for High Entropy Relaxor Ferroelectric (Li_0.2_Ca_0.2_Sr_0.2_Ba_0.2_La_0.2_)TiO_3_ Ceramic. J. Alloys Compd..

[41] Braga M.H., Murchison A.J., Oliveira J.E., Goodenough J.B. (2019). Low-Temperature Performance of a Ferroelectric Glass Electrolyte Rechargeable Cell. ACS Appl. Energy Mater..

[42] Braga M.H. (2021). Coherence in the Ferroelectric A_3_ClO (A = Li, Na) Family of Electrolytes. Materials.

[43] Li S., Wang F., Wang Y., Yang J., Wang X., Zhan X., He J., Wang Z. (2024). Van Der Waals Ferroelectrics: Theories, Materials, and Device Applications. Adv. Mater..

[44] Liu S., Kim Y., Tan L.Z., Rappe A.M. (2016). Strain-Induced Ferroelectric Topological Insulator. Nano Lett..

[45] Gomes B.M., Moutinho J.F.R., Braga M.H. (2024). A Perspective on the Building Blocks of a Solid-State Battery: From Solid Electrolytes to Quantum Power Harvesting and Storage. J. Mater. Chem. A Mater..

[46] Monserrat B., Bennett J.W., Rabe K.M., Vanderbilt D. (2017). Antiferroelectric Topological Insulators in Orthorhombic A MgBi Compounds (A = Li, Na, K). Phys. Rev. Lett..

[47] Dutt A., Minkov M., Williamson I.A.D., Fan S. (2020). Higher-Order Topological Insulators in Synthetic Dimensions. Light. Sci. Appl..

[48] Braga M.H. (2024). Energy Harnessing and Storage from Surface Switching with a Ferroelectric Electrolyte. Chem. Commun..

[49] Gandi S.S., Katta V.K., Jayasankar C.K., Pecharapa W., Ravuri B.R. (2023). Glass-Ceramic Na_3+x_[(Zr/Cr)_x_(Sc/Ti)_2−x_(PO_4_)_3_ Electrolyte Materials for Na-Ion Full-Cell Application. Integr. Ferroelectr..

[50] Gopinadh S.V., Anoopkumar V., Ansari M.J.N., Srivastava D., Raj M.A., John B., Samridh A., Vijayakumar P.S., Mercy T.D. (2022). Lithium-Ion Pouch Cells: An Overview. Energy Harvesting and Storage: Fundamentals and Materials.

[51] Wang X., Zhang Q., Zhao C., Li H., Zhang B., Zeng G., Tang Y., Huang Z., Hwang I., Zhang H. (2024). Achieving a High-Performance Sodium-Ion Pouch Cell by Regulating Intergrowth Structures in a Layered Oxide Cathode with Anionic Redox. Nat. Energy.

[52] Tang J., Barker J., Pol V.G. (2018). Sodium-ion Battery Anodes Comprising Carbon Sheets: Stable Cycling in Half-and Full-pouch Cell Configuration. Energy Technol..

[53] Lim G.J.H., Chan K.K., Sutrisnoh N.A.A., Srinivasan M. (2022). Design of Structural Batteries: Carbon Fibers and Alternative Form Factors. Mater. Today Sustain..

[54] Parviziomran E., Elliot V. (2024). Barriers to Circular Economy: Insights from a Small Electric Vehicle Battery Manufacturer. J. Purch. Supply Manag..

[55] Chigbu B.I. (2024). Advancing Sustainable Development through Circular Economy and Skill Development in EV Lithium-Ion Battery Recycling: A Comprehensive Review. Front. Sustain..

[56] Zhao T., Mahandra H., Marthi R., Ji X., Zhao W., Chae S., Traversy M., Li W., Yu F., Li L. (2024). An Overview on the Life Cycle of Lithium Iron Phosphate: Synthesis, Modification, Application, and Recycling. Chem. Eng. J..

[57] Hu G., Huang K., Du K., Peng Z., Cao Y. (2024). Efficient Recovery and Regeneration of FePO4 from Lithium Extraction Slag: Towards Sustainable LiFePO4 Battery Recycling. J. Clean. Prod..

[58] Gucciardi E., Galceran M., Bustinza A., Bekaert E., Casas-Cabanas M. (2021). Sustainable Paths to a Circular Economy: Reusing Aged Li-Ion FePO4 Cathodes within Na-Ion Cells. J. Phys. Mater..

[59] Wesselkämper J., Dahrendorf L., Mauler L., Lux S., Von Delft S. (2024). Towards Circular Battery Supply Chains: Strategies to Reduce Material Demand and the Impact on Mining and Recycling. Resour. Policy.

[60] Carvalho Baptista M., Khalifa H., Araújo A., Maia B.A., Souto M., Braga M.H., Baptista M.C., Khalifa H., Maia B.A., Braga M.H. (2022). Giant Polarization in Quasi-Adiabatic Ferroelectric Na^+^ Electrolyte for Solid-State Energy Harvesting and Storage. Adv. Funct. Mater..

[61] Kresse G., Furthmüller J. (1996). Efficient Iterative Schemes for Ab Initio Total-Energy Calculations Using a Plane-Wave Basis Set. Phys. Rev. B Condens. Matter Mater. Phys..

[62] Baptista M.C., Gomes B.M., Vale A.B., Braga M.H. (2024). In-Series All-Solid-State Anode-Less Cells. J. Energy Storage.

[63] Wang S., Zhang J., Gharbi O., Vivier V., Gao M., Orazem M.E. (2021). Electrochemical Impedance Spectroscopy. Nat. Rev. Methods Primers.

[64] Lazanas A.C., Prodromidis M.I. (2023). Electrochemical Impedance Spectroscopy—A Tutorial. ACS Meas. Sci. Au.

[65] Vadhva P., Hu J., Johnson M.J., Stocker R., Braglia M., Brett D.J.L., Rettie A.J.E. (2021). Electrochemical Impedance Spectroscopy for All-solid-state Batteries: Theory, Methods and Future Outlook. ChemElectroChem.

[66] Rusling J.F., Suib S.L. (1994). Characterizing Materials with Cyclic Voltammetry. Adv. Mater..

[67] Morales D.M., Risch M. (2021). Seven Steps to Reliable Cyclic Voltammetry Measurements for the Determination of Double Layer Capacitance. J. Phys. Energy.

[68] Newbury D.E., Ritchie N.W.M. (2013). Is Scanning Electron Microscopy/Energy Dispersive X-ray Spectrometry (SEM/EDS) Quantitative?. Scanning.

